# Correction: PMA-qPCA: Accelerating the market release of high-quality *Bradyrhizobium diazoefficiens* inoculant

**DOI:** 10.1371/journal.pone.0343418

**Published:** 2026-02-18

**Authors:** Mariana Cap, Camila Frydman, Antonella Galiñanes, Camila Aranguiz, Irina Faraco, Luisina Andriolo, Viviana Parreño, Marina Mozgovoj

The method “PMA-qPCR” is misspelled in the article title. The correct title is: PMA-qPCR: Accelerating the market release of high-quality *Bradyrhizobium diazoefficiens* inoculant. The correct citation is: Cap M, Frydman C, Galiñanes A, Aranguiz C, Faraco I, Andriolo L, et al. (2025) PMA-qPCR: Accelerating the market release of high-quality *Bradyrhizobium diazoefficiens* inoculant. PLoS One 20(10): e0325878. https://doi.org/10.1371/journal.pone.0325878.

There is an error in the Funding statement. The correct Funding statement is as follows: This study was funded by private funds from Rizobacter S.A. and public funds from the research project 2023-PE-L04-I120 of the National Institute of Agricultural Technology.
