# Supplementary material for: PMA-qPCA: Accelerating the market release of high-quality Bradyrhizobium diazoefficiens inoculant
Source: PLoS One. 2025 Oct 21;20(10):e0325878. doi: 10.1371/journal.pone.0325878 (PMC12539688; doi:10.1371/journal.pone.0325878)
Supplement: Attachment — Submitted filename: PONE.docx [file pone.0325878.s008.docx]

Dr. Mohammad H. Ghazimoradi

The manuscript presents a well-designed study validating the use of PMA-qPCR for rapid quantification of viable *Bradyrhizobium diazoefficiens* in inoculants, offering a significant improvement over the traditional culture-based method. The work is technically sound, addresses a relevant agricultural challenge, and demonstrates robust experimental validation. However, some clarifications and additional details would strengthen the manuscript.

Best regards,

Maryam Zakavi

Major Comments

1. Methodological Clarity
   - PMA Optimization (Section 2.5.1): The manuscript states that 50 µM PMA was selected for subsequent assays but does not explicitly justify why this concentration was optimal (e.g., statistical comparison of inhibition efficacy across 50, 75, and 100 µM). Include a quantitative analysis (e.g., % signal inhibition at each concentration) to support this choice.
   - Heat Inactivation Controls: Clarify whether heat inactivation (95°C for 10 min) was validated to ensure complete cell death without DNA fragmentation, which could affect PMA binding efficiency. A viability stain (e.g., SYTOX Green) could corroborate this.
2. Statistical Analysis
   - Figure 2c (Experiment 4): The inability to distinguish 2-fold dilutions contrasts with the stated SD of 0.3 log CFU/mL. Reconcile this discrepancy by discussing technical limitations (e.g., pipetting error, PMA heterogeneity) or proposing a higher dilution factor for finer discrimination.
   - Regression Analysis (Figure 3a): The R² of 0.82 suggests unexplained variability. Discuss potential sources (e.g., matrix effects, PMA penetration variability) and how they might be mitigated in industrial applications.
3. Comparative Advantages
   - Time/Cost Savings: Emphasize the economic impact of reducing processing time from 120 h to 5 h. Include a brief cost comparison (e.g., reagents, labor) between PMA-qPCR and plate counting to highlight scalability.
   - VBNC State: The dismissal of VBNC cells (lines 259–261) lacks experimental evidence. Cite supporting data or reference prior studies showing cryoprotectants prevent VBNC formation in *Bradyrhizobium*.
4. Industrial Applicability
   - Sample Throughput: Describe how the method scales for high-throughput batch testing (e.g., number of samples processed per run, automation potential).
   - Matrix Variability: Address whether the method was tested across different inoculant formulations (e.g., peat-based vs. liquid carriers) to ensure broad applicability.

Minor Comments

1. Abstract
   - Replace "82% correlation" with "R² = 0.82" for statistical precision.
2. Introduction
   - Line 52: Clarify how PMA’s nitrene radical binds DNA (covalent vs. intercalation) to aid reader understanding.
3. Results
   - Figure 1: Label axes in all panels (e.g., "Log CFU/mL" for bacterial counts).
   - Line 165: Specify which model (beta-Poisson or exponential) yielded the LOD of 3.14 log CFU/mL.
4. Discussion
   - Line 226: Expand on why LOD/LOQ values are acceptable despite being higher than other applications (e.g., inoculants typically have high cell densities).
5. References
   - Ensure all citations (e.g., Pedrolo et al., 2024) are in the bibliography.

Recommendation

Accept with Minor Revisions pending clarification of the above points. The study’s innovation and practical utility for inoculant quality control are compelling, but addressing these issues will enhance rigor and readability.

Additional Suggestion: Consider a supplemental table summarizing key performance metrics (e.g., LOD, LOQ, CV%, R²) for quick reference by readers.

Reviewer Confidence: High (the methodology aligns with established PMA-qPCR protocols, and results are well-supported by data).
